# Thermal Analysis of Radiative Darcy–Forchheimer Nanofluid Flow Across an Inclined Stretching Surface

**DOI:** 10.3390/nano12234291

**Published:** 2022-12-02

**Authors:** Jifeng Cui, Ahmed Jan, Umer Farooq, Muzamil Hussain, Waseem Asghar Khan

**Affiliations:** 1 College of Science, Inner Mongolia University of Technology, Hohhot 010051, China; 2 Department of Mathematics, COMSATS University Islamabad, Park Road, Tarlai Kalan, Islamabad 45550, Pakistan; 3 Department of Mathematics, University of the Poonch Rawalakot, Rawalakot 12350, Pakistan; 4 Department of Mathematics, Faculty of Sciences AlZulfi, Majmaah University, Majmaah 11952, Saudi Arabia

**Keywords:** Casson nanofluid, inclined stretching surface, Darcy–Forchheimer model, non-similarity, bvp4c

## Abstract

Nanofluids have unique features that make them potentially valuable in a variety of medicinal, technical, and industrial sectors. The widespread applications of nanotechnology in modern science have prompted researchers to study nanofluid models from different perspectives. The objective of the current research is to study the flow of non-Newtonian nanofluid over an inclined stretching surface immersed in porous media by employing the Darcy–Forchheimer model. Both titanium oxide ($TiO_{2}$) and aluminum oxide ($Al_{2}O_{3}$) are nanoparticles which can be found in blood (based fluid). The consequences of viscous dissipation, thermal radiations, and heat generation are also incorporated. Boundary layer approximations are employed to model the governing system of partial differential equations (PDEs). The governing PDEs with their associated boundary conditions are further altered to a dimensionless form by employing appropriate transformations. The results of the transformed model are collected using local non-similarity approach up to the second level of truncation in association with the built-in finite difference code in MATLAB (bvp4c). Additionally, the impacts of emerging factors on the fluid flow and thermal transport features of the considered flow problem are displayed and analyzed in graphical forms after achieving good agreement between accomplished computational results and published ones. Numerical variations in drag coefficient and Nusselt number are elaborated through the tables. It has been perceived that the enhancement in Casson fluid parameter diminishes the velocity profile. Moreover, it is noted that the porosity parameter and Lorentz’s forces reinforce the resulting frictional factor at the inclined stretching surface.

## 1. Introduction

Turning nanoparticles into colloidal dispersions in liquid media, which is part of a new classification of heat transmission in fluids, creates what is known as nanofluids in today’s nanotechnology era. Nanoparticles such as metals, metal oxides, etc., are frequently employed as nanofluids with a solid phase due to their efficient benefits such as low density, chemical stability, and simplicity of preparation. The physically and chemically interlinked features of nano-sized particles and molecule-based nanofluids play a considerable role in the viscosity and thermal conductivity of colloidal dispersion solution. Thus, nano-sized engineering of fluids can ensure the thermal and physical features of nanofluids. Due to the wide range of technological applications that enable diverse industrial heat transfer processes, researchers’ interest in studying nanofluids is growing at the moment. Mahian et al. [1] presented a theoretical and computational examination of the heat transfer and entropy minimization of nanofluid flows across diverse flow regimes and geometries. Esfe et al. [2] addressed thermal system optimization because an improved energy system has numerous benefits such as reduced operating costs, limited negative environmental impacts, increased heat transfer coefficient, and energy source savings. Chahregh and Dinarvand [3] developed an effective mathematical model for the transport of nanofluid in a porous channel using *Ag* and *TiO_2_* as nanoparticles and blood as the base fluid, which may be an appropriate drug delivery model. Chiolerio et al. [4] studied the effects of ferrofluids on the transport processes which can be controlled by varying the strength and orientation of an external magnetic field. Bhatti et al. [5] assessed the effects of porosity, magnetic field, and motile microbe suspension on 3-dimensional, incompressible, axisymmetric, unstable Williamson nanofluid flow between two parallel rotating plates with its potential wide range of industrial applications. Pordanjani et al. [6] presented a critical assessment of the uses of nanofluids in various thermal systems and their environmental repercussions. With activation energy and chemical reaction assumptions, Shahid et al. [7] investigated bio-convection impacts on MHD Carreau nanofluid flow through the paraboloid porous surface. Ali et al. [8] investigated the effects of nanometer-sized copper (*Cu*) particles in water (base fluid) with the considerations of non-linear thermal radiations, variable fluid viscosity, Joule heating, viscous dissipation, and non-uniform heat flux.

Non-Newtonian nanofluids may be observed in a wide range of commercial and technical applications, including dissolved polymers, biological solutions, asphalts, glues, and paints. Therefore, the dynamics of non-Newtonian nanofluids have attracted the interest of many academics. Since a single constitutive equation cannot fully capture the physical characteristics of non-Newtonian fluids, several models have been developed to do so. Casson fluid is one of these non-Newtonian fluids. With a yield shear stress included in the constitutive equation, it exhibits behavior similar to that of elastic solids; jelly, honey, soup, tomato sauce, and concentrated fruit liquids are all examples of Casson fluids. Blood in certain situations can also behave as Casson fluid. Several studies on blood with different hematocrits, anticoagulants, temperatures, etc., suggest that blood behaves as a Casson fluid. In particular, the Casson fluid model more accurately depicts the flow characteristics of blood at low shear rates. Several recent investigations regarding Casson nanofluid flow can be found in Refs. [9,10,11]. A study on the boundary layer of Maxwell nanofluid with induced magnetic field and analysis of entropy for unsteady viscoelastic fluids was also conducted by Asjad et al. [12]. The effect of temperature on the temperature-dependent thermal conductivity and viscosity of Williamson nanofluid flow over an exponentially stretching permeable vertical plate was examined by Amjad et al. [13]. Hussain et al. [14] presented a novel analysis regarding 2-dimensional electromagnetohydrodynamic Casson nanofluid flow with thermal radiations, and heat source/sink impacts across a stretching surface by considering iron oxide and gold as nanoparticles. Zari et al. [15] scrutinized the heat and mass transfer with the effects of double stratification for the Marangoni Casson convective nanofluid flow across an inclined surface. 

The science of magnetohydrodynamics (MHD) examines how an electrically conducting fluid moves in a magnetic field, which can influence the system’s heat transfer flow. Theoretically, magnetic fields induce a drag force known as the Lorentz force, which resists the flow and thus increases the temperature distribution of the fluid. Nevertheless, the applied magnetic field complicates the separation of the boundary layer. MHD has several applications in crystal development, metal casting, optical grafting, tunable optical fiber filters, MHD generators, and the polymer sector. Hartmann and Lazarus [16] were the pioneers who established the hypothesis of an electrically conducting laminar fluid flow in a homogeneous magnetic field, launching massive research efforts in MHD throughout the previous few decades. Anusha et al. [17] investigated the influence of a magnetic field across the stretching surface integrated with porous media on the 2-dimensional laminar couple stress water-based nanofluid flow. With the presumptions of zero nanoparticle flux and first-order compound response, Dey and Mukhopadhyay [18] investigated the impacts of an external magnetic field and suction/injection on forced convection nanofluid flow across an absorbent surface. Evaristo et al. [19] investigated the unsteady MHD model to simulate the process of heat transfer which goes through temperature and magnetic field cycles to generate mechanical power. Arulmozhi et al. [20] assessed the influences of heat radiation and chemical reactions on the MHD nanofluid’s mass and heat convection over an infinitely moving vertical surface embedded in porous media. Jan et al. [21] examined the MHD boundary-layer nanofluid flow over a vertically placed stretching surface under the impacts of viscous dissipation and heat generation/absorption factor. Vishalakshi et al. [22] examined the non-Newtonian three-dimensional graphene water nanofluid flow across a porous stretching/shrinking surface in the presence of thermal radiation and mass transpiration. 

Porous surfaces and media play a crucial part in engineering, agricultural, and industrial fields such as drying procedures, oil recovery, geothermal energy, chromatography, fuel cell equipment, etc. An active method for enhancing thermal performance is the cumulative impact of heat and mass transfer associated with the MHD flow of nanofluids across a porous media. In a variety of technical and industrial processes, fluid velocity and thermal management are successfully regulated in porous media. Darcy presented a classical concept to simulate flow through a porous media. However, this notion holds for analyses with lower velocity and lower porosity. On the other side, when nanofluids flow at a faster pace than the conventional Darcy theory is inappropriate, an extended Darcy–Forchheimer model is employed to characterize various fluid flows. Wang et al. [23] examined thermal transportation associated with the flow of non-Newtonian nanofluid triggered by a stretching surface across the Darcy–Forchheimer medium with the consideration of electromagnetic field. Bilal et al. [24] analyzed the nanofluid flow comprising magnesium oxide (MgO), titanium dioxide (TiO_2_) and cobalt ferrite (CoFe_2_O_4_) nanoparticles through plate, wedge, and cone with the consequences of activation energy, heat source/sink, and the magnetic field. Rasool et al. [25] considered the Buongiorno’s model to examine the consequences of the thermal radiations and Darcy–Forchheimer medium on the MHD Maxwell nanofluid flow across a stretching surface. Eswaramoorthi et al. [26] scrutinized the radiative Darcy–Forchheimer flow of Casson nanofluids across a stretching surface with suction and heat consumption. 

The growing usage of non-Newtonian nanofluids in a variety of biological and technical applications have prompted to examine steady incompressible non-Newtonian nanofluid flow across an inclined stretching surface through porous media. The non-Newtonian Casson fluid model is considered for flow analysis. The nanofluid comprises nanoparticles such as $Al_{2}O_{3}$, and $TiO_{2}$, and blood as the base fluid. The consequences of thermal radiations, heat production, and viscous dissipation are also considered by using the (Tiwari and Das) single-phase nanofluid model. The governing system is transformed into a non-similar arrangement by employing appropriate conversions. The transformed equations are tackled using local non-similarity (LNS) technique [27] via the bvp4c solver provided in the computational MATLAB software. To the best of the authors’ knowledge, no similar studies on this topic have yet been investigated. The stimulus of dimensionless emerging factors on velocity and thermal profiles are discussed thoroughly with graphs. In addition, the consequences of several dimensionless parameters on surface drag coefficient and heat transfer characteristics are also investigated.

## 2. Mathematical Modeling

**Figure 1:**
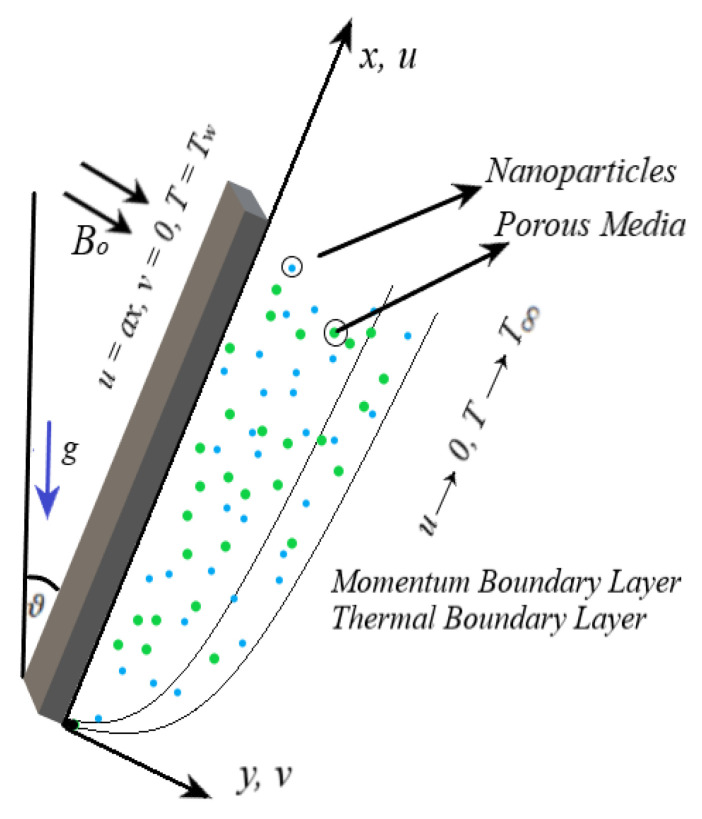
Flow configuration.

Consider the steady incompressible magnetized flow of Casson nanofluid contains nanoparticles of $\mathrm{titanium}\;\mathrm{oxide}\left(TiO_{2}\right)$ and $\mathrm{aluminum}\;\mathrm{oxide}\left(Al_{2}O_{3}\right)$ dispersed in base fluid (blood) across an inclined stretching surface with the assumption of a Darcy-Forchheimer porous medium. The inclined stretching surface stretches with the velocity $u_{w}\left(x\right)$, and has temperature $T_{w},$ whereas the ambient velocity is assumed to be zero and the ambient temperature is $T_{\infty }$. Nanofluid flow is electrically conductive. The applied magnetic field $B_{o}$ is placed normal to the stretching surface (Figure 1). The impacts of heat source/sink factor and thermal radiations are also considered. Incorporating Boussinesq, and boundary layer assumptions the basic conservation equations of mass, momentum, and energy are given, respectively, by [9,10,11]:

(1) $$\frac{\partial u}{\partial x}+\frac{\partial v}{\partial y}=$$

(2) $$\rho_{nf}\left(u\frac{\partial u}{\partial x}+v\frac{\partial u}{\partial y}\right)=\mu_{nf}\left(1+\frac{1}{\beta }\right)\left(\frac{\partial^{2}u}{\partial y^{2}}\right)+{\left(\rho \beta_{T}\right)}_{nf}gcos\vartheta \left(T-T_{\infty }\right)-\frac{\mu_{nf}}{K}u-\sigma_{nf}B_{0}^{2}u-F_{o}u^{2},\;$$

(3) $$\begin{array}{l} {\left(\rho c_{p}\right)}_{nf}\left(u\frac{\partial T}{\partial x}+v\frac{\partial T}{\partial y}\right)\\ \;\;\;\;\;=k_{nf}\frac{\partial^{2}T}{\partial y^{2}}+\mu_{nf}\left(1+\frac{1}{\beta }\right){\left(\frac{\partial u}{\partial y}\right)}^{2}+\sigma_{nf}{\left(uB_{o}\right)}^{2}\\ \;\;\;\;\;+\frac{16}{3}\left(\frac{\sigma^{*}T_{\infty }^{3}}{k^{*}}\frac{\partial^{2}T}{\partial y^{2}}\right)+\frac{\mu_{nf}}{K}u^{2}+Q_{o}\left(T-T_{\infty }\right). \end{array}$$

With boundary conditions:

(4) $$u=u_{w}\left(x\right)=bx,v=0\;,\;T=T_{w}\;,\;\;\;\;\;\;at\;y=0$$

(5) $$u=0=v,\;\;\;\;\;T\to T_{\infty \;\;}\;\;at\;y\to \infty .$$

Here u and v are the velocity vectors in x- and y-directions, β is the Casson fluid parameter, K (permeability of porous medium), $F_{o}=\frac{C_{b}}{xK^{\frac{1}{2}}}$ (non-uniform inertial coefficient), where $C_{b}$ is the drag force coefficient, $B_{o}$ (strength of magnetic field), $Q_{o}$ (rate of heat generation /absorption), $k^{*}$ (Boltzmann constant), fluid density is ρ, $C_{p}$, $T_{\infty \;}$ and $T_{w}$ specifies the specific heat, ambient, and wall temperatures. 

To develop a non-similar flow, introducing the ξ(x) as non-similarity, and η(y) as a pseudo-similarity variable.

(6) $$\xi =\frac{x}{l},\;\;\eta =y\sqrt{\frac{b}{\nu_{nf}}},\;\;u=bx\frac{\partial f}{\partial \eta }\left(\xi ,\eta \right),\;v=-\sqrt{b\nu_{nf}}\left(f\left(\xi ,\eta \right)+\xi \frac{\partial f}{\partial \xi }\left(\xi ,\eta \right)\right),\;\theta \left(\;\xi ,\eta \right)=\frac{\left(T-T_{\infty }\right)}{\left(T_{w}-T_{\infty }\right)}.$$

In the light of the above transformations, Equations (6) and (1) are identically satisfied, whereas Equations (2)–(5) become:

(7) $$\begin{array}{l} \left(1+\frac{1}{\beta }\right)\frac{\partial^{3}f}{\partial \eta^{3}}+\left[f\frac{\partial^{2}f}{\partial \eta^{2}}-\left(1-F_{r}\right){\left(\frac{\partial f}{\partial \eta }\right)}^{2}\right]\\ \;\;\;\;\;\;-\frac{\rho_{nf}}{\rho_{f}}\left[\frac{\mu_{f}}{\mu_{nf}}\lambda \frac{\partial f}{\partial \eta }+\frac{\sigma_{nf}}{\sigma_{f}}M\frac{\partial f}{\partial \eta }-\frac{{\left(\rho \beta_{T}\right)}_{nf}}{{\left(\rho \beta_{T}\right)}_{f}}\xi^{-1}G_{r}\theta cos\vartheta \right]\\ \;\;\;\;\;\;=\xi \left(\frac{\partial f}{\partial \eta }\frac{\partial^{2}f}{\partial \xi \partial \eta }-\frac{\partial f}{\partial \xi }\frac{\partial^{2}f}{\partial \eta^{2}}\right) \end{array}$$

(8) $$\begin{array}{l} \left(\frac{k_{nf}}{k_{f}}+R_{d}\right)\frac{\partial^{2}\theta }{\partial \eta^{2}}\\ \;\;\;\;\;+EcPr\frac{\mu_{nf}}{\mu_{f}}[\left(1+\frac{1}{\beta }\right){\left(\frac{\partial^{2}f}{\partial \eta^{2}}\right)}^{2}+\xi^{2}\frac{\sigma_{nf}}{\sigma_{f}}M{\left(\frac{\partial f}{\partial \eta }\right)}^{2}\\ \;\;\;\;\;+\xi^{2}\lambda \frac{\mu_{nf}}{\mu_{f}}{\left(\frac{\partial f}{\partial \eta }\right)}^{2}]+\frac{\mu_{nf}}{\mu_{f}}\frac{{\left(\rho c_{p}\right)}_{nf}}{{\left(\rho c_{p}\right)}_{f}}Prf\frac{\partial \theta }{\partial \eta }\\ \;\;\;\;\;+\frac{\mu_{nf}}{\mu_{f}}PrQ\theta =\frac{\mu_{nf}}{\mu_{f}}\frac{{\left(\rho c_{p}\right)}_{f}}{{\left(\rho c_{p}\right)}_{nf}}Pr\xi \left(\frac{\partial f}{\partial \eta }\frac{\partial \theta }{\partial \xi }-\frac{\partial f}{\partial \xi }\frac{\partial \theta }{\partial \eta }\right) \end{array}$$

Non-similar boundary conditions are:

(9) $$\frac{\partial f}{\partial \eta }\left(\xi ,0\right)=1,\;f\left(\xi ,0\right)+\xi \frac{\partial f\left(\xi ,0\right)}{\partial \xi }=0,\;\theta \left(\xi ,0\right)=1,\;$$

(10) $$\frac{\partial f\left(\xi ,\infty \right)}{\partial \eta }=0,\;\theta \left(\xi ,\infty \right)=0$$

where the inertia coefficient is $F_{r}=\frac{C_{b}}{K^{\frac{1}{2}}}$. magnetic number $M=\frac{\sigma_{f}B_{o}^{2}}{b\rho_{f}}$, porosity parameter $\lambda =\frac{\nu_{f}}{bk\;}$, mixed convection parameter $G_{r}=\frac{g\beta_{f}\left(T_{w}-T_{\infty }\right)}{b^{2}l}$, radiation factor $Rd=\frac{16}{3}\frac{\sigma^{*}T_{\infty }^{3}}{k^{*}k_{f}}$, Eckert number $Ec=\frac{c^{2}l^{2}}{{\left(c_{p}\right)}_{f}\left(T_{w}-T_{\infty }\right)}$ and Prandtl number $Pr=\frac{\nu_{f}}{\alpha_{f}}$, heat source/sink factor $Q={\frac{Q_{o}}{b\left(\rho c_{p}\right)}}_{f}$. 

Surface friction coefficient $C_{f}$ and local Nusselt number $Nu_{x}$ are:

(11) $$C_{f}={\left(\frac{\tau_{w}}{\rho_{nf}{\left(u_{w}\right)}^{2}}\right)}_{y=0},\;N={\left(\frac{xq_{w}}{k_{f}\left(T_{w}-T_{\infty }\right)}\right)}_{y=0}$$

where $\tau_{w}$ surface shear stress, $q_{w}$ is the surface flux:

(12) $$\tau_{w}=\mu_{nf}{\left(\frac{\partial u}{\partial y}\right)}_{y=0},\;q_{w}=-{\left[k_{nf}\frac{\partial T}{\partial y}+q_{r}\right]}_{y=0}$$

Using Equation (5) and Equation (10), we have:

(13) $$\begin{array}{c} Re^{\frac{1}{2}}C_{f}=\xi^{-1}\frac{\partial^{2}f\left(\xi ,0\right)}{\partial \eta^{2}}\\ Re^{\frac{-1}{2}}Nu=-\xi \left(\frac{k_{nf}}{k_{f}}+Rd\right)\frac{\partial \theta \left(\xi ,0\right)}{\partial \eta } \end{array}\}$$

The Reynold’s number is defined as $Re=\frac{bl^{2}}{\nu_{f}}$.

### 2.1. Solution Methodology

The dimensionless governing model mentioned in Equations (7)–(10) for boundary-layer flow of nanofluid across an inclined stretching surface is solved by employing the methodology of LNS. The stepwise details of the LNS method for the stated problem are in the next subsections.

### 2.2. Local Non-Similarity Method: 

Assuming that the terms $\xi \frac{\partial \left(.\right)}{\partial \xi }$ are very small at the first level of truncation, and this behavior is true for (ξ << 1). Therefore, Equations (7)–(10) become:

(14) $$\begin{array}{l} \left(1+\frac{1}{\beta }\right)f^{\prime \prime \prime }=\left(1-F_{r}\right){\left(f^{\prime }\right)}^{2}-ff^{\prime \prime }\\ \;\;\;\;\;\;-\frac{\rho_{f}}{\rho_{nf}}\left[-\frac{\mu_{f}}{\mu_{nf}}\lambda f^{\prime }-\frac{\sigma_{nf}}{\sigma_{f}}Mf^{\prime }-\frac{{\left(\rho \beta_{T}\right)}_{nf}}{{\left(\rho \beta_{T}\right)}_{f}}\xi^{-1}G_{r}\theta cos\vartheta \right], \end{array}$$

(15) $$\begin{array}{l} \left(\frac{k_{nf}}{k_{f}}+R_{d}\right)\theta^{\prime \prime }\\ \;\;\;\;\;+EcPr\frac{\mu_{nf}}{\mu_{f}}[\left(1+\frac{1}{\beta }\right){f^{\prime \prime }}^{2}+\frac{\sigma_{nf}}{\sigma_{f}}\xi^{2}M{f^{\prime }}^{2}\\ \;\;\;\;\;+\xi^{2}\lambda \frac{\mu_{nf}}{\mu_{f}}{f^{\prime }}^{2}]+\frac{\mu_{nf}}{\mu_{f}}PrQ\theta +\frac{\mu_{nf}}{\mu_{f}}\frac{{\left(\rho c_{p}\right)}_{nf}}{{\left(\rho c_{p}\right)}_{f}}Prf\theta^{\prime }\\ \;\;\;\;\;=0. \end{array}$$

The accompanying boundary conditions are:

(16) $$f^{\prime }\left(\xi ,0\right)=1,\;f\left(\xi ,0\right)=0,\;\theta \left(\xi ,0\right)=1,$$

(17) $$f^{\prime }\left(\xi ,\infty \right)=0,\;\theta \left(\xi ,\infty \right)=0.$$

To obtain second-order truncation, Equations (7)–(10) must be differentiated with respect to ξ and the introduction of new functions $g\left(\xi ,\eta \right)=\frac{\partial f\left(\xi ,\eta \right)}{\partial \xi },\;h\left(\xi ,\eta \right)=\frac{\partial \theta \left(\xi ,\eta \right)}{\partial \xi }$, and equating the ξ-derivatives to reach zero, such that $\frac{\partial g\left(\xi ,\eta \right)}{\partial \xi }=\frac{\partial h\left(\xi ,\eta \right)}{\partial \xi }=0$. The transformed equations are:

(18) $$\begin{array}{l} \left(1+\frac{1}{\beta }\right)g^{\prime \prime \prime }=\left[f^{\prime }g^{\prime }-gf^{\prime \prime }+\xi \left({g^{\prime }}^{2}-gg^{\prime \prime }\right)\right]\\ \;\;\;\;\;\;-\left[gf^{\prime \prime }+fg^{\prime \prime }-2\left(1-F_{r}\right)f^{\prime }g^{\prime }\right]\\ \;\;\;\;\;\;+\frac{\rho_{nf}}{\rho_{f}}[\frac{\mu_{f}}{\mu_{nf}}\lambda g^{\prime }+\frac{\sigma_{nf}}{\sigma_{f}}Mg^{\prime }\\ \;\;\;\;\;\;+\frac{{\left(\rho \beta_{T}\right)}_{nf}}{{\left(\rho \beta_{T}\right)}_{f}}\left(\xi^{-2}G_{r}\theta cos\vartheta -\xi^{-1}G_{r}hcos\vartheta \right)] \end{array}$$

(19) $$\begin{array}{c} \left(\frac{k_{nf}}{k_{f}}+R_{d}\right)\theta^{\prime \prime }\\ \;\;\;\;\;=\frac{\mu_{nf}}{\mu_{f}}EcPr\left[2\left(1+\frac{1}{\beta }\right)f^{\prime \prime }g^{\prime \prime }+2\frac{\sigma_{nf}}{\sigma_{f}}M\xi {\left(f^{\prime }\right)}^{2}\right.\\ \;\;\;\;\;+2\frac{\sigma_{nf}}{\sigma_{f}}M\xi^{2}f^{\prime }g^{\prime }\\ \;\;\;\;\;\left.+\lambda \frac{\mu_{nf}}{\mu_{f}}\frac{{\left(\rho c_{p}\right)}_{f}}{{\left(\rho c_{p}\right)}_{nf}}\left(\xi {\left(f^{\prime }\right)}^{2}+\xi^{2}f^{\prime }g^{\prime }\right)\right]-\frac{\mu_{nf}}{\mu_{f}}PrQh\\ \;\;\;\;\;-\frac{\mu_{nf}}{\mu_{f}}\frac{{\left(\rho c_{p}\right)}_{nf}}{{\left(\rho c_{p}\right)}_{f}}Pr\left[g\theta^{\prime }+fh^{\prime }\right]\\ \;\;\;\;\;+\frac{\mu_{nf}}{\mu_{f}}\frac{{\left(\rho c_{p}\right)}_{f}}{{\left(\rho c_{p}\right)}_{nf}}Pr\left[f^{\prime }h-g\theta^{\prime }+\frac{\mu_{nf}}{\mu_{f}}\xi \left(g^{\prime }h-gh^{\prime }\right)\right] \end{array}$$

subjected to the conditions:

(20) $$g^{\prime }\left(\xi ,0\right)=0,\;\;\;\;g\left(\xi ,0\right)=0,\;\;h\left(\xi ,0\right)=0,$$

(21) $$g^{\prime }\left(\xi ,\infty \right)=0,\;h\left(\xi ,\infty \right)=0$$

Table 1 represents thermophysical properties of important variables of nanofluids.

**Table 1:** Thermophysical properties of the nano fluid [28].

Property	Symbol	Defined
Viscosity	$\mu_{nf}$	$\mu_{nf}=\frac{\mu_{f}}{{\left(1-\phi \right)}^{2.5}}$
Density	$\rho_{nf}$	$\rho_{nf}=\left(1-\phi \right)\rho_{f}+\phi \rho_{s}$
Heat capacitance	${\left(\rho C_{p}\right)}_{nf}$	${\left(\rho C_{p}\right)}_{nf}=\left(1-\phi \right){\left(\rho C_{p}\right)}_{f}+\phi {\left(\rho C_{p}\right)}_{s}$
Electric conductivity	$\sigma_{nf}$	$\sigma_{\mathrm{nf}}=\left\{1+\frac{3\left(\frac{\sigma_{s}}{\sigma_{f}}-1\right)\phi }{\left(\frac{\sigma_{s}}{\sigma_{f}}+2\right)-\left(\frac{\sigma_{s}}{\sigma_{f}}-1\right)\phi }\right\}\sigma_{f}$
Thermal conductivity	$k_{nf}$	$k_{nf}=\frac{\left(k_{s}+2k_{f}\right)-2\phi \left(k_{f}-k_{s}\right)}{\left(k_{s}+2k_{f}\right)+\phi \left(k_{f}-k_{s}\right)}k_{f}$
Thermal expansion	${\left(\rho \beta_{T}\right)}_{nf}$	${\left(\rho \beta_{T}\right)}_{nf}=\left(1-\phi \right){\left(\rho \beta_{T}\right)}_{f}+\phi {\left(\rho \beta_{T}\right)}_{s}$

Where ϕ is the concentration of the nanoparticles.

Table 2 represents thermophysical properties of nanoparticles and base fluids.

**Table 2:** Thermophysical values of nanoparticles and base fluid [29].

Materials	$c_{p}$ (J/kgK)	ρ (kg/m^3^)	k(W/mK)	β(1K)	$\sigma (\Omega m){\;}^{-1}$
***Titanium Oxide*** **(*TiO_2_*)**	686.2	4250	8.9538		$1.0\times 10^{-12}$
***Aluminum Oxide*** **(*Al_2_O_3_*)**	765	3970	40.0	8.5 $\times 10^{-6}$	$1.0\times 10^{-10}$
***Blood***	3594	1053	0.492	0.18 $\times 10^{-5}$	0.8

Furthermore, the comparative study with the existing literature has been displayed in Table 3 (see [30,31,32]). This shows that the present findings are quite similar as compared to the results in existing literature. It proves that the presented solutions are valid in limiting case.

Table 4 and Table 5 deliberate the response of the drag force coefficient and Nusselt number for different values of emerging parameters. It is noticed in Table 4 that, the values of $Re^{\frac{1}{2}}C_{f}$ elevate with $M,\;G_{r}$, and λ, whereas they reduces with increasing levels of β.

Findings in Table 5 show that $Re^{-\frac{1}{2}}Nu$ varies directly with (M) and (Q), but has an inverse relation with λ, Ec, β, and Rd, taking ξ=0.1. 

## 3. Result and Discussion

In this section, the physical discussion is described on the graphs that are developed to examine the behavior of various dimensionless physical parameters against the velocity and temperature profiles. A comparison has been shown in each graph for two different nanofluids, namely, $TiO_{2\;}$+ blood and $Al_{2}O_{3}\;$+ blood. 

**Figure 2:**
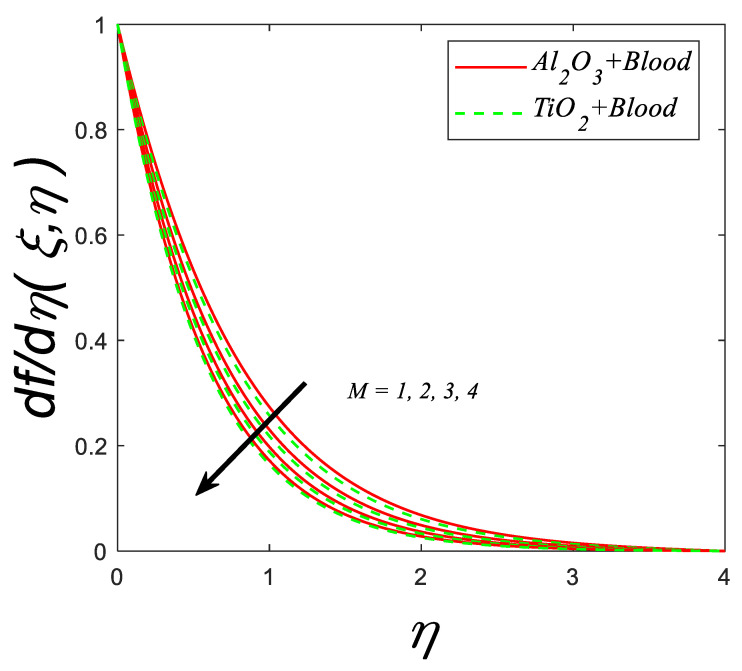
Velocity profile for deviating estimations of M. When $Q=0.1,\vartheta =\frac{\pi }{3},\;\xi =0.1,Rd=0.1,\;Gr=0.1,\;Pr=21,\;Ec=0.1,\;\lambda =0.5,\;\beta =1.5,\;\phi =0.1$.

The predicted consequence of the magnetic number M on fluid velocity is shown in Figure 2. The interaction of magnetic force reflects a diminishing change in velocity due to the Lorentz’s force existence.

**Figure 3:**
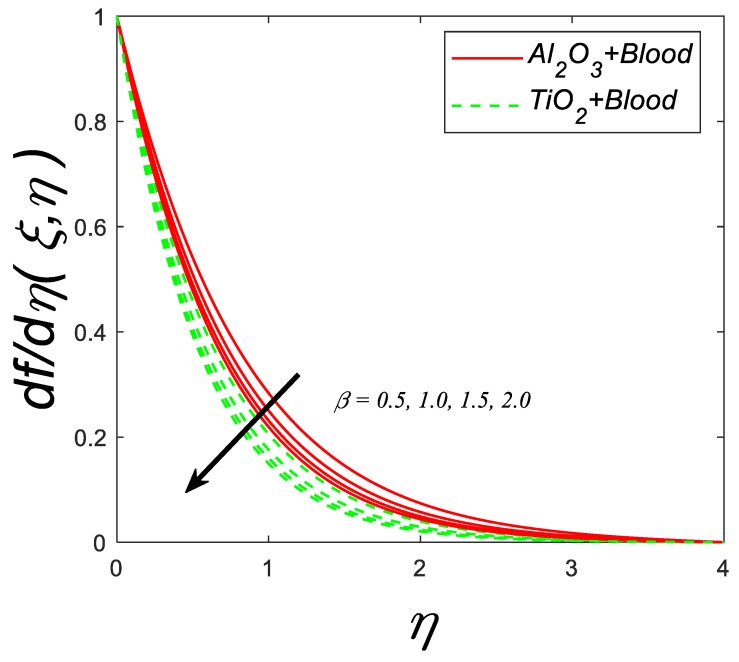
Velocity profile for deviating estimations of β. When $Q=0.1,\;\vartheta =\frac{\pi }{3},\;\xi =0.1,Rd=0.1,\;Gr=0.1,\;Pr=21,\;Ec=0.1,\;\lambda =0.5,M=0.3,\;\phi =0.1$.

Figure 3 depicts the velocity profile against the Casson fluid parameter (β). The graph shows that as (β) increases, there is a decline in the velocity profile. Physically, it is anticipated since an upsurge in β causes an increase in the fluid’s dynamic viscosity. This triggers a decline in velocity profile owing to the development of fluid viscosity.

**Figure 4:**
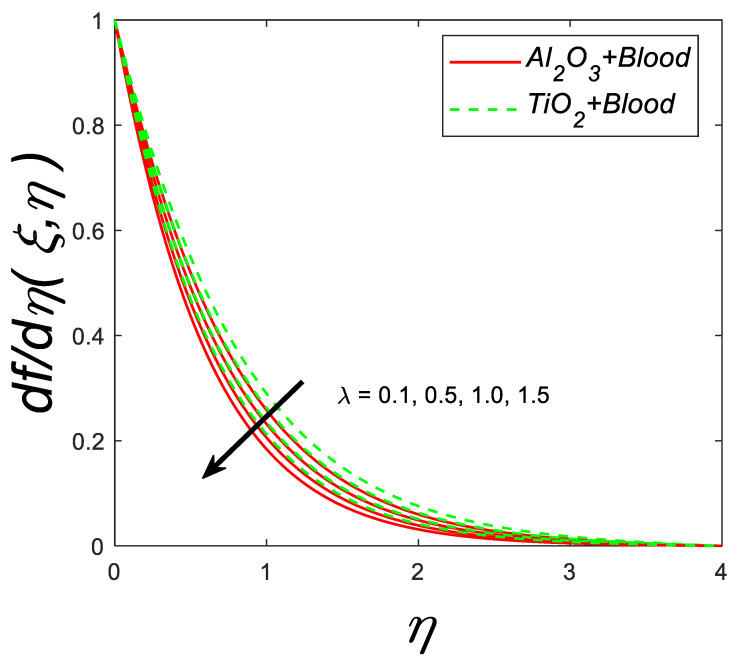
Velocity profile for deviating estimations of λ. When $Q=0.1,\;\vartheta =\frac{\pi }{3},\;\xi =0.1,Rd=0.1,\;Gr=0.1,\;Pr=21,\;Ec=0.1,\;M=0.3,\;\beta =1.5,\;\phi =0.1$.

The impact of porosity parameter (λ) against velocity profile is clear from Figure 4. An increment in (λ) shows the decline in the velocity profile. Physically, this conclusion can be examined that with the enhancement in λ, the viscosity of the fluid increases therefore, velocity decrease.

**Figure 5:**
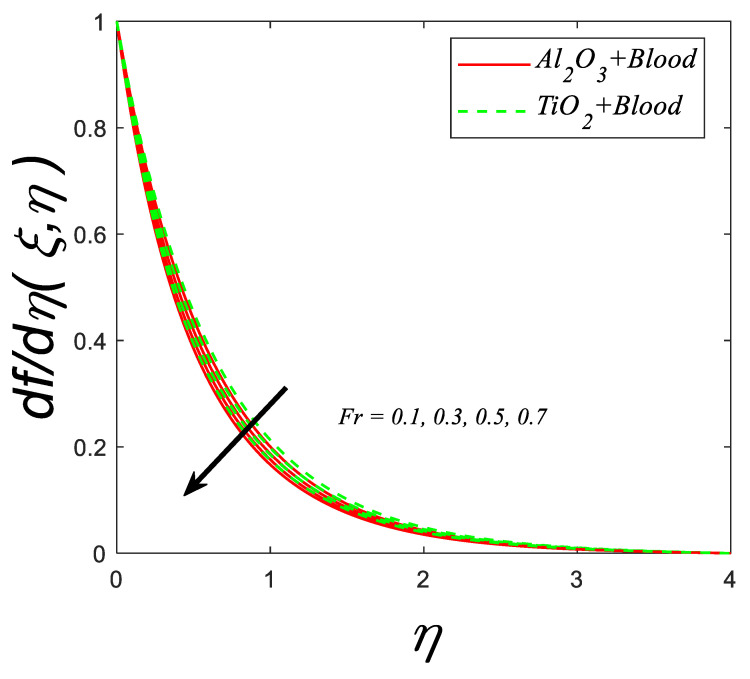
Velocity profile for deviating estimations of $F_{r}$. When $Q=0.1,\;\vartheta =\frac{\pi }{3},\;\xi =0.1,\;Rd=0.1,\;Gr=0.1,\;Pr=21,\;Ec=0.1,\;\lambda =0.5,\;\beta =1.5,\;\phi =0.1,\;M=0.3$.

Figure 5 depicts the fluctuation of the velocity profile against the Forchheimer number $\left(F_{r}\right)$. The graph shows that the increasing $\left(F_{r}\right)$ slows decline in the fluid velocity. When the coefficient of inertia is overestimated, fluid velocity decreases. The inertia coefficient is associated with drag force in this case. Although fluid velocity drops as the inertia coefficient grows, so does the drag force.

**Figure 6:**
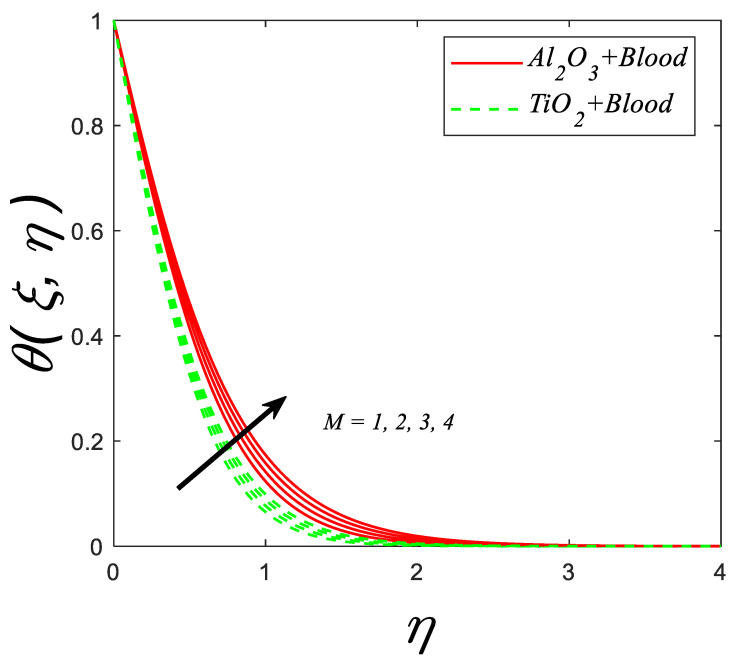
Temperature profile for deviating estimations of M. When $Q=0.1,\;\vartheta =\frac{\pi }{3},\;\xi =0.1,Rd=0.1,\;Gr=0.1,\;Pr=21,\;Ec=0.1,\;\lambda =0.5,\;\beta =1.5,\;\phi =0.1$.

Figure 6 depicts how the magnetic parameter (M) affects the thermal profile. (M) causes the temperature of the nanoparticles to rise. It is physically justified that, with the increasing (M), the Lorentz force linked with the magnetic field strengthens and it generates more resistance to the flow which leads to the enhancement in thermal boundary-layer thickness.

**Figure 7:**
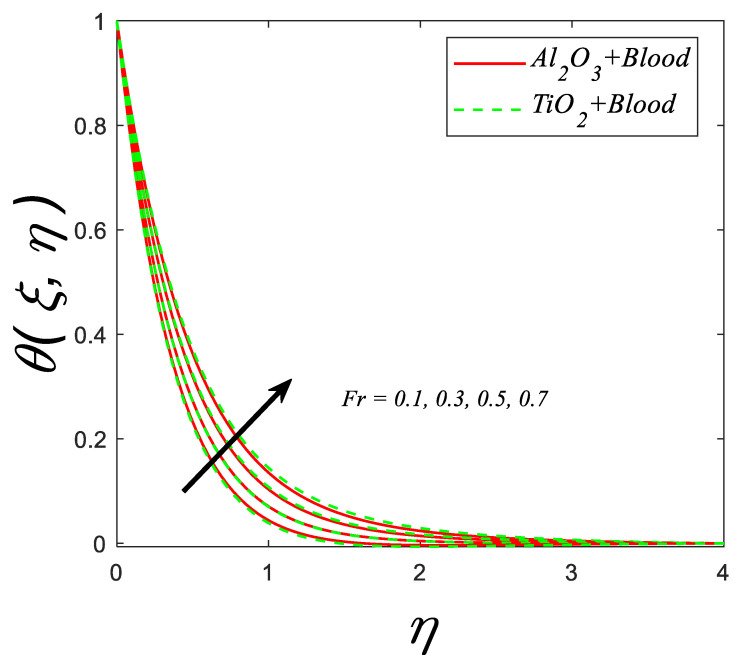
Temperature profile for deviating estimations of Fr. When $M=0.3,Q=0.1,\;\vartheta =\frac{\pi }{3},\;\xi =0.1,\;Rd=0.1,\;Gr=0.1,\;Pr=21,\;Ec=0.1,\;\lambda =0.5,\;\beta =1.5,\;\phi =0.1$.

Figure 7 indicates the consequence of the inertial coefficient $\left(F_{r}\right)$ against the thermal profile. The graph illustrates that increasing the inertia coefficient results in a significant improvement in the temperature profile. Because the inertia coefficient correlates directly with porosity media and drag coefficient, a high value of $C_{b}$ increases both media porosity and drag coefficient; thus, the velocity decreases against $\left(F_{r}\right)$, whereas the fluid temperature increases.

**Figure 8:**
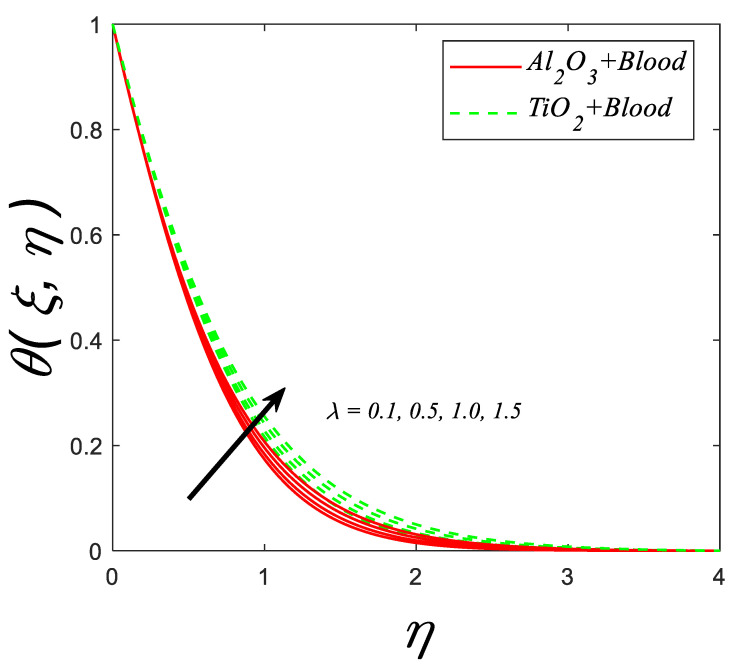
Temperature profile for deviating estimations of λ. When $Q=0.1,\;\vartheta =\frac{\pi }{3},\;Rd=0.1,\;Gr=0.1,\;\xi =0.1,\;Pr=21,\;Ec=0.1,M=0.3,\;\beta =1.5,\;\phi =0.1$.

The impact of porosity parameter (λ) against the temperature profile is shown in Figure 8. With an increment in (λ), an escalation in viscosity occurs which enhance the thermal profile.

**Figure 9:**
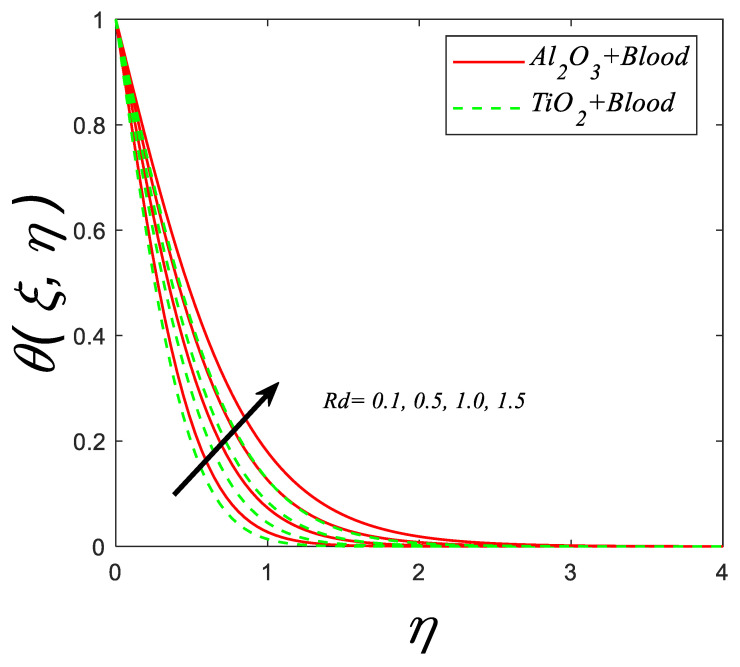
Temperature profile for deviating estimations of $R_{d}$. When $Q=0.1,\;\vartheta =\frac{\pi }{3},M=0.3,\;\xi =0.1,\;Gr=0.1,\;Pr=21,\;Ec=0.1,\;\lambda =0.5,\;\beta =1.5,\;\phi =0.1$.

Figure 9 exhibits the behavior of the temperature profile against the various estimations of the radiation parameter $\left(R_{d}\right)$. For increasing values of $\left(R_{d}\right)$, temperature profile increases for both cases.

**Figure 10:**
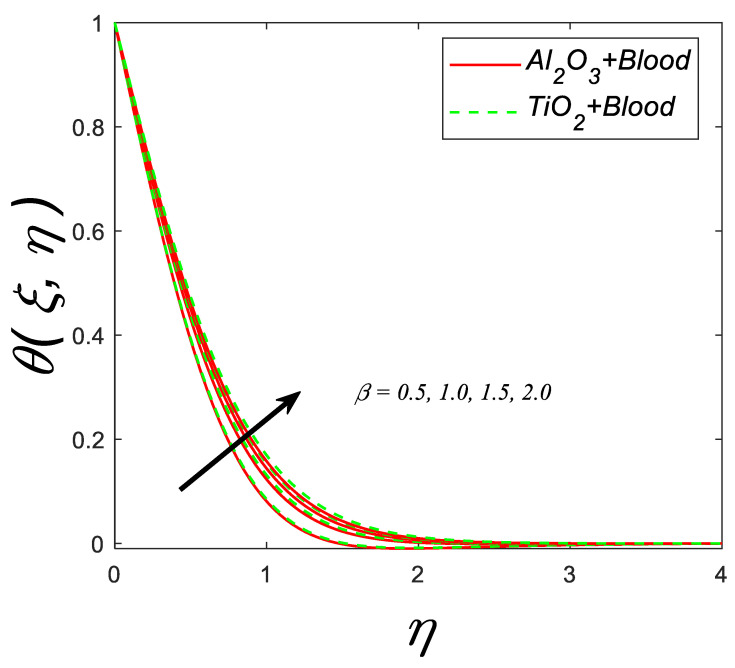
Temperature profile for deviating estimations of β. When $Q=0.1,\vartheta =\frac{\pi }{3},\;\xi =0.1,\;Rd=0.1,\;Gr=0.1,\;Pr=21,\;Ec=0.1,\;\lambda =0.5,\;M=0.3,\;\phi =0.1$.

The behavior of the temperature profile in response to the variations in the Casson parameter (β) is shown in Figure 10, as the Casson parameter (β) enhances the thermal boundary layer enhances.

**Figure 11:**
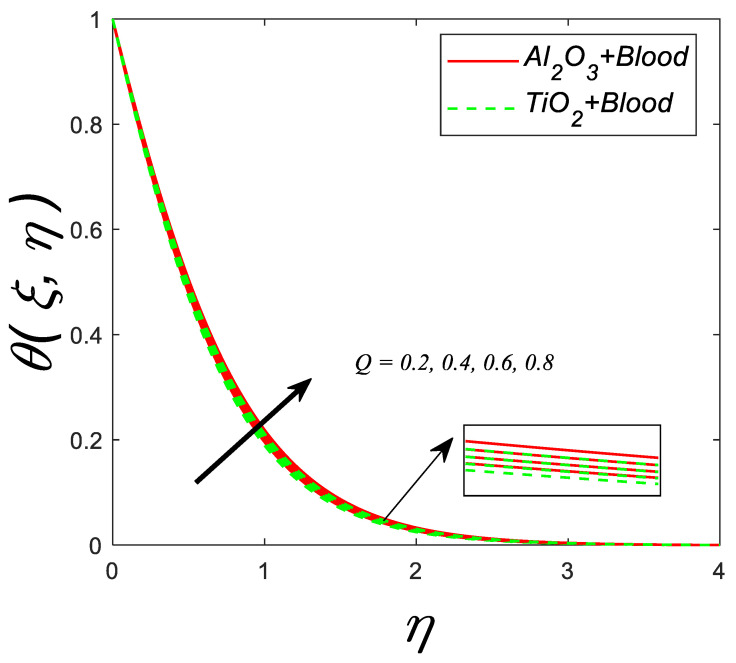
Temperature profile for deviating estimations of Q. When $Q=0.1,\;\vartheta =\frac{\pi }{3},\;\xi =0.1,\;Rd=0.1,\;Gr=0.1,\;Pr=21,\;Ec=0.1,\;\lambda =0.5,\;\beta =1.5,\;\phi =0.1$.

Figure 11 describes the behavior of thermal profile for various estimations of heat source/sink parameter (Q). Enhancement in (Q), (Q>0) significantly accelerates the thermal profile.

## 4. Conclusions

In the considered problem, non-similar analysis for Casson nanofluid flow over an inclined permeable stretching surface is proposed with the impacts of heat generation, thermal radiations, magnetic field, viscous dissipation, and porous medium using the Darcy–Forchheimer model. The governing system is highly non-linear and is successfully tackled by using the LNS approach in association with the bvp4c MATLAB package. The following are the key conclusions of the present research:

⮚ The flow is decelerated with increasing estimations of the magnetic number and the Casson parameter.
⮚ By enhancing the magnetic number M, the flow field decreases while the thermal profile increases.
⮚ Additionally, the thermal profile rises as the porosity parameter is estimated to be higher, and the flow field also decreases.
⮚ Thermal profiles are significantly increased with radiation parameters.
⮚ An increment in the porosity parameter and the Eckert number reduces the local Nusselt number, whereas they increase the thermal boundary layer thickness for both considered cases.
⮚ A greater magnetic parameter (stronger Lorentz force) enhances the magnitude of the drag coefficient.
⮚ To validate the existing analysis, a comparative study is conducted, which proves the consistency of the current results.

## Figures and Tables

**Table 3:** Comparison of the $\theta^{\prime }\left(0\right)$ when ξ=0.1 and $F_{r}=M=G_{r}=Ec=Q=Rd=0\;$ with published literature Refs. [30,31,32].

*Pr*		$\theta^{\prime }\left(0\right)$		
	Hassanien et al. [30]	Salleh et al. [31]	Alkasasbeh et al. [32]	Present Study
0.72	0.46325	0.46317	0.46316	0.4986316451
1	0.58198	0.58198	0.58198	0.5975809450
3	1.16525	1.16522	1.16524	1.1643563783
5		1.56806	1.56807	1.5674514653
7		1.89548	1.89550	1.8949925136
10	2.30801	2.30821	2.30820	2.3153556715
100	7.74925	7.76249	7.76250	7.7714379105

**Table 4:** Computed values of $Re^{\frac{1}{2}}C_{f}$ against several estimations of M, β, λ, and Gr when ξ=0.1.

M	$G_{r}$	λ	β	$\left(Al_{2}O_{3}\right)$ + Blood	$\left(TiO_{2}\right)$ + Blood
0.2	0.1	0.2	1.5	0.4901010947	0.4274598638
0.3				0.5475697381	0.4345869467
0.2	0.1			‒0.3248568971	‒0.2776837564
	0.2			‒0.2785986793	‒0.2185968970
		0.2		0.5353017252	0.4509675312
		0.3		0.6140624653	0.4766629609
			1.5	0.5673852317	0.2543942356
			2.0	0.5379535782	0.2348687475

**Table 5:** Computed values of $Re^{-\frac{1}{2}}Nu$ against several values of β, M, Q *,* Rd *,* Ec, and Pr when ξ=0.1.

M	λ	Ec	β	Rd	Q	$\left(Al_{2}O_{3}\right)$ + Blood	$\left(TiO_{2}\right)$ + Blood
0.1	0.1	0.01	1.5	0.1	0.2	1.6224248090	1.6875579845
0.3						1.5786434551	1.6468293505
0.1	0.1					1.6224248090	1.6875579845
	0.2					1.4359703456	1.4687930578
		0.01				1.6224248090	1.6875579845
		0.03				1.5783456731	1.6134984516
			1.5			1.6224248090	1.6875579845
			2.0			1.6203188133	1.6234887611
				0.1		1.6224248090	1.6875579845
				0.3		1.5643867971	1.6436739765
					0.1	1.6224248090	1.6875579845
					0.3	1.7659238752	1.7535962614

## Data Availability

Not applicable.

## Nomenclature

u, v	Velocity components (*ms^−1^*)
x,y	Coordinate System (*m*)
T	Temperature (*K*)
$T_{w}$	Surface temperature (*K*)
$T_{\infty }$	Ambient temperature (*K*)
ρ	Fluid density (*kg/m^−3^*)
α	Thermal diffusivity (*m^2^/s*)
$F_{o}$	Non-uniform inertial coefficient
$F_{r}$	Inertial coefficient
$\beta_{T}$	$\mathrm{Thermal}\;\mathrm{expansion}\;\left(K^{-1}\right)$
β	Casson fluid parameter
$C_{b}$	Drag force
$k^{*}$	Boltzmann constant
ϑ	Angle of inclination
K	Permeability of porous medium
$Q_{0}$	Heat generation/absorption coefficient
f	Dimensionless stream function
θ	Dimensionless temperature
η	Pseudo-similarity variable
ξ	Non-similarity variable
M	Magnetic parameter
$G_{r}$	Grashof number
Rd	Radiation parameter
Re	Reynolds number
Pr	Prandtl number
λ	Porosity parameter
Ec	Eckert number
f, nf	Base fluid, nano fluid
$B_{0}$	Magnetic parameter(*kgs^−2^A^−1^*)
k	Thermal conductivity (*W/mK*)
$c_{p}$	Specific heat(*m^2^s−^−2^k^−1^*)
σ	Electrical conductivity (*kg^−1^m^−3^s^3^A^2^*)
$C_{f}$	Skin friction coefficient
Nu	Local Nusselt number
$\tau_{w}$	Surface shear stress (*kgm^−1^s−^−2^*)
